
JOURNAL INFORMATION
==============================
Journal ID: Wirtschaftsdienst

Wirtschaftsdienst

EISSN: 1613-978X

ARTICLE INFORMATION
==============================
PMCID: PMC9584269
DOI: 10.1007/s10273-022-3303-4
Article ID: 3303
Article version: 1
Subjects: Zeitgespräch

Entlastungspaket: Stabilisierung der Erwartungen

Relief Package: Stabilisation of Expectations

Hüther Michael huether@iwkoeln.de

grid.473597.b 0000 0000 9854 4639 Institut der deutschen Wirtschaft Köln e.V., Konrad-Adenauer-Ufer 21, 50668 Köln, Deutschland
Electronic publication date: 2022 Oct 20
Print publication date: 2022
Volume: 102
Issue: 10
First page: 757
Last page: 760
Copyright: © Der/die Autor:in 2022
License: Open Access: Dieser Artikel wird unter der Creative Commons Namensnennung 4.0 International Lizenz veröffentlicht (https://creativecommons.org/licenses/by/4.0/deed.de).
Open Access wird durch die ZBW — Leibniz-Informationszentrum Wirtschaft gefördert.
License URL: https://creativecommons.org/licenses/by/4.0/

Keywords: H12, H20, H50
issue-copyright-statement: © The Author(s) 2022

==============================

Die Bundesregierung (2022a) hat sich Anfang September 2022 auf ein drittes Entlastungspaket geeinigt, um die Folgen der importierten Teuerung für die privaten Haushalte, aber auch kleine und mittlere Unternehmen abzufedern. Derzeit (Anfang Oktober 2022) ist nicht abzusehen, was in welcher Dimension umgesetzt werden wird, da die Bundesländer grundsätzlich zustimmen müssen. Unabhängig davon stellen sich grundsätzliche Fragen für eine angemessene Wirtschaftspolitik in dieser Krise und die konkrete Ausgestaltung denkbarer Instrumente:

- Um was für eine Krise handelt es sich bzw. welches Krisenverständnis können wir angesichts der extremen Unsicherheit zugrunde legen? Wie groß ist das Risiko, dass die Krise ihre Qualität verändert?
- Welche wirtschaftspolitischen Instrumente – überwiegend im Sinne der Prozesspolitik – mit welcher Intensität und Zeitlichkeit wirken angemessen für die so gedeutete Lage und Entwicklungsperspektive?
- Welche wirtschaftspolitischen Maßnahmen, die aus anderen Gründen – ordnungs- und strukturpolitischen – geboten sind, entfalten in diesem Kontext eine positive Wirkung?

## Risiko der Deindustrialisierung

Das Ausmaß der Krise hat sich im Laufe des Jahres zunehmend deutlicher erkennen lassen. Aus dem Schock nach dem Beginn des russischen Überfalls auf die Ukraine ist anfänglich die Einschätzung abgeleitet worden, es könne eine vergleichbare ökonomische Herausforderung wie die Pandemie sein, also befristet auf ein bis zwei Jahre. Auch der Begriff der Rezession, der im Laufe des Jahres 2022 zunehmend in den Prognosen und öffentlich-medial an Bedeutung gewonnen hat, deutet auf ein entsprechend zeitlich begrenztes Phänomen hin.

Tatsächlich aber bergen die kräftig gestiegenen Energiepreise ein erhebliches strukturelles Risiko für die deutsche Volkswirtschaft: Die Gaspreiskrise wirkt zusammen mit einer auch originären Strompreiskrise über Kostensteigerung sowie Kaufkraftentzug und ist zu einer Überforderungskrise geworden, und zwar sowohl bei den liquiditätsbeschränkten privaten Haushalten als auch in vielen Branchen der Volkswirtschaft. Denn es erhöhen sich nicht nur vorübergehend die Produktionskosten, da eine Substitution von Gas in vielen Fällen schwierig ist und Zeit braucht. Hinzu kommt die fehlende Planbarkeit, da niemand mit Gewissheit sagen kann, inwieweit die Gasversorgung in den kommenden anderthalb Jahren gesichert ist.

In den ersten beiden Kriegsmonaten ist der Preis für Erdgas um mehr als 400 % gestiegen. Dies befeuert die Inflation – 60 % der Inflationsrate sind auf Energierohstoffe und Energiedienstleistungen zurückzuführen. Zudem verursacht der Gaspreis über die Merit Order im Strommarkt im Zusammenspiel mit der dort fehlenden gesicherten Kapazität eine entsprechende kräftige und erratische Entwicklung des Strompreises. Diese Steigerung der Energiekosten macht deutlich, dass die politische Aussage, während der Energiewende seien durchgängig Versorgungssicherheit und Wettbewerbsfähigkeit gewährleistet, sich in einer Stresssituation wie derzeit als falsch erweist.

So erleben wir anders als in der Pandemie, der Weltfinanzkrise oder grundsätzlich allen früheren Rezessionen, dass diesmal die energieintensive Grundstoffproduktion als Erste unter massiven Anpassungsdruck steht. Dabei handelt sich es um Branchen, die aufgrund der hohen Industriestrompreise ohnehin schon seit längerem ihren Kapitalstock in Deutschland verringern, aber unverändert für viele industrielle Wertschöpfungsketten hierzulande eine zentrale Bedeutung haben. Das hat vor allem mit der Materialforschung und deren Rolle in vielen zukunftsträchtigen Verwendungen zu tun. Es zeigen sich in energieintensiven Branchen seit Kriegsbeginn bereits erhebliche Produktionseinschränkungen (vgl. Abbildung 1).

Angesichts der Krisendimension, die sich deutlich von den beiden vorangegangenen Schocks unterscheidet, ist die Herausforderung der Wirtschaftspolitik, möglichst an den Ursachen anzusetzen, ebenso dringlich wie schwierig. Denn in einer Kriegswirtschaft, und damit haben wir

Abbildung 1

**Produktionsrückgang im Verarbeitenden Gewerbe, Deutschland**

Index 1/2021 = 100

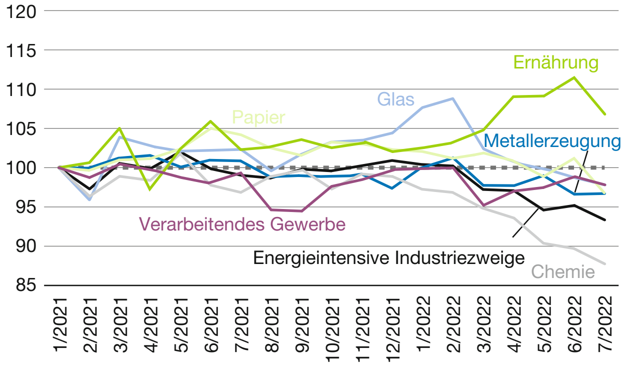

Quelle: Macrobond, Statistisches Bundesamt, eigene Berechnungen.

Tabelle 1

**Stilisierte Darstellung denkbarer Szenarien für die deutsche Volkswirtschaft**

| Szenario 1: Rezession (2023/24) | Szenario 2: Deindustrialisierung (bis 2030) |
|---|---|
| Inflation geht im Laufe 2023 spürbar zurück | Inflation bleibt zunächst hoch |
| Unternehmen können stabilisiert werden | Insolvenzwelle |
| Keine Deindustrialisierung | Deindustrialisierung ausgehend von der Grundstoffproduktion |
| Re-Start für aktive Transformation | Passive Transformation |
| Kein Einbruch am Arbeitsmarkt | Massenarbeitslosigkeit |
| Stabilisierung des privaten Verbrauchs | Nachfragedeflation |

Quelle: eigene Darstellung.

es faktisch zu tun, geht es um Mengenrationierung; in diesem Fall zunächst bei Gas. Das hat dazu geführt, dass der Gasmarkt völlig in Unordnung geraten ist, politische Preise unabhängig von den Fundamentaldaten dominieren und wegen der angespannten Angebotslage die Liquidität massiv eingeschränkt ist.

Die Politik müsste ursachentherapeutisch einerseits alles tun, um das Angebot auszuweiten. Das ist durch die Bundesregierung mit den Beschaffungsbemühungen im Rahmen der kurzfristigen Möglichkeiten geleistet worden; vor allem das Auffüllen der Gasspeicher auf über 90 % schon im September steht dafür. Allerdings hat die Einspeicherung zunächst den Markt illiquide gemacht, und kann erst entspannend wirken, wenn gezielt im Winterhalbjahr entspeichert wird. Andererseits muss versucht werden Anreize für das Einsparen von Gas zu setzen. Bisher ist dies vor allem von Seiten der Industrie durch die Produktionseinschränkungen und Substitutionsanstrengungen erreicht worden. Die Reaktion der Endkund:innen (private Haushalte) steht noch aus. In Europa kann man feststellen, dass in den Ländern (z. B. Niederlande), wo die Preissignale ganz oder überwiegend wirksam waren, die Einsparungen sehr deutlich sind, und dort (z. B. Frankreich), wo die Preiseffekte für die Endkund:innen neutralisiert wurden, keine Mengeneinschränkungen festzustellen sind (Oxford Economics, 2022).

Jenseits dieser Kombination aus Beschaffung von Gas und der Wirksamkeit von Sparanreizen für die Gaskund:innen wird derzeit intensiv darüber diskutiert, wie die Kostenüberforderung der Unternehmen und der privaten Haushalte über Eingriffe in die Preisbildung am Gasmarkt und am Strommarkt vermindert werden kann. Beide Überlegungen finden sich im dritten Entlastungspaket, freilich nur als nicht konkretisierte Ankündigung und unter Vorbehalt gemeinsamer europäischer Lösungen. Am 29. September wurde in der Bundesregierung (2022b) ein Kompromiss für die Finanzierung des Strompreis- und Gaspreisdeckels gefunden, indem dafür der Wirtschaftsstabilisierungsfonds mit Kreditermächtigungen von 200 Mrd. Euro ausgestattet wird. Die konkrete Ausgestaltung der Gaspreis- oder Gastkostenbremse soll den Vorschlägen der entsprechenden Kommission folgen.

Die Politik hat damit anerkannt, dass wir es mit einer tiefgehenden Strukturkrise zu tun haben, die den Kern des deutschen Geschäftsmodells – die Industrie – bedroht. Stilisiert lassen sich zwei Szenarien für die weitere Entwicklung beschreiben: Eine Rezession mit dem üblichen begrenzten Zeitprofil versus eine lange anhaltende Strukturkrise, an deren Ende eine Deindustrialisierung steht (vgl. Tabelle 1).

In welche Richtung sich die tatsächliche Entwicklung bewegt, wird wesentlich davon abhängen, ob es kurzfristig gelingt, die Kosteneffekte bei den Unternehmen und den Kaufkraftentzug bei den privaten Haushalten so abzufedern, dass sie über den Winter kommen. In dieser Zeit muss es gelingen, aufgrund der Beschlüsse von Ende September positive Erwartungen für die Energiekosten auf mittlere Sicht durch Eingriffe am Gasmarkt und am Strommarkt zu begründen.

## Stabilisierung der privaten Haushalte und der Unternehmen: Liquidität gesucht

Damit rücken Überlegungen zu kurzfristig angemessenen wirtschaftspolitischen Maßnahmen – also im Sinne der Prozesspolitik – in den Mittelpunkt. Die Bundesregierung hatte bereits in den ersten Entlastungspaketen mit unterschiedlichen Instrumenten wie dem Heizkostenzuschuss, Kinderbonus und Energiepreispauschale vor allem die privaten Haushalte adressiert, die liquiditätsbeschränkt sind. Beide Pakete haben zusammen ein Volumen von gut 30 Mrd. Euro. Aus der Kritik an den Paketen erklären sich einige Maßnahmen, die jetzt gesetzlich über das dritte Paket auf den Weg gebracht werden sollen. Dazu zählen vor allem die Einmalzahlungen für Rentner:innen, Studierende sowie die Aussicht auf eine Wohngeldreform mit dauerhafter Heizkosten- und Klimakomponente.

Bei der Unterstützung für Unternehmen, die durch die Energiekosten überfordert oder gar existentiell bedroht sind, ist die Politik bislang sehr zögerlich gewesen. Die bestehenden Instrumente – vor allem das Bund-Länder-Bürgschaftsprogramm und das Energiekostendämpfungsprogramm – adressieren Bedarfslagen in energieintensiven Branchen, beim Handel mit Energie und andere Betroffenheiten, die aus dem Krieg resultieren. Über Haftungsfreistellung wird versucht, Kredite leichter verfügbar zu machen, und es werden Zuschüsse zu den Energiekosten im Volumen von 5 Mrd. Euro angeboten. Aber das reicht bei weitem nicht aus. Denn die tatsächliche Lage differiert zwischen Unternehmen je nach Branche, Region und Energieabhängigkeit so stark, dass antragsabhängige Lösungen übermäßig verwaltungs-, kontroll- und damit zeitaufwendig sind. Es bedarf aber einer schnellen Liquiditätsstützung. Dafür ist auch in den Beschlüssen vom 29. September 2022 nichts zu finden.

Es bietet sich eine steuerpolitische Lösung an (Hentze und Hüther, 2022): Sie ist über die Finanzämter schnell umsetzbar, wo die Konto- und Steuerdaten der Unternehmen schon vorliegen. Sie begünstigt ausschließlich Unternehmen, die aufgrund ihrer bisherigen Performance Steuern zahlen, und sie belastet den Staatshaushalt durch eine Verschiebung der Einnahmen zunächst nur temporär. Der Grundgedanke lautet: Angesichts der fundamental veränderten Lage in den Kostenstrukturen der Unternehmen sowie – im binnenwirtschaftlichen Bereich – wegbrechender Konsumnachfrage, beruhen die Vorauszahlungen in der Einkommen-, der Gewerbe-, der Körperschaft- sowie der Umsatzsteuer auf Annahmen, die mittlerweile unrealistisch sind. Deshalb wäre zu überlegen, allen Unternehmen die anstehenden Vorauszahlungen für das 4. Quartal 2022, das 1. Quartal 2023 sowie längstens das 2. Quartal 2023 auf die Körperschaft-, Einkommen-, Gewerbe- und Umsatzsteuer automatisch, also ohne Antrag, zu stunden. Ein Verzicht auf die Prüfung von Kriterien und Kennzahlen sorgt für unbürokratische Entlastung bei Unternehmen und Verwaltung.

Zudem sollte für besonders belastete Unternehmen auf Antrag eine Rückzahlung der geleisteten Steuervorauszahlungen für das 2. und 3. Quartal 2022 möglich sein. Zu dieser Gruppe gehören Unternehmen, die einen überdurchschnittlichen Anteil an Energiekosten haben (in Anlehnung an bestehende Hilfsprogramme mindestens 3 % Energiekostenanteil am Umsatz des Jahres 2021) oder stark unter dem Rückgang des Konsums leiden (etwa ein Umsatzrückgang von Juli bis September 2022 um 30 % gegenüber Vorjahr).

Schließlich könnte die im Rahmen der Coronapandemie erweiterte Verlustverrechnung für alle Unternehmen in drei Dimensionen erweitert werden: Die Gewerbesteuer wird in die Verlustverrechnung einbezogen, der Verlustrücktrag gilt der Höhe nach unbegrenzt und der Zeitraum zu seiner Nutzung wird auf drei Jahre erweitert. In der Coronapandemie wurde der Verlustrücktrag auf zwei Jahre ausgedehnt, seine Grenze liegt befristet bis Ende 2023 bei 10 Mio. Euro. Auch die Begrenzung des Verlustvortrags von 1 Mio. Euro pro Jahr sollte gelockert werden.

Kapitalgesellschaften, Personengesellschaften und Einzelunternehmer sowie Selbstständige sollten und können gleichermaßen einbezogen werden. Die Stundung eines Quartals hätte bei den Ertragssteuern maximal einen Liquiditätseffekt von grob gerechnet 40 Mrd. Euro, bei der Umsatzsteuer von rund 50 Mrd. Euro. Hinzu kämen antragsabhängig durch die Erstattung gezahlter Vorauszahlungen weitere Liquiditätshilfen. Der temporäre Aufkommenseffekt könnte gesteuert werden, indem Stundung oder Erstattung von Vorauszahlungen nach Steuerart und Unternehmensgröße eingeschränkt werden.

Zu beachten ist, dass es sich in allen Fällen dem Grunde nach um rückzahlbare Darlehen des Staates an die steuerpflichtigen Unternehmen handelt. Nach Maßgabe der wirtschaftlichen Lage der Unternehmen wird rückblickend die definitive Steuerlast für das Jahr 2022 festgestellt. Darauf bezogen wird der Staatshaushalt kaum zusätzlich belastet. Europarechtliche Probleme dürften nicht auftreten. Klar ist aber, dass diese Hilfen nur den Übergang in das Jahr 2023 begleiten können, nicht aber die Knappheit bei Gas und die preislichen Herausforderungen im Strommarkt lösen werden.

### Warum der Abbau der kalten Progression keine Steuerentlastung ist

Verglichen mit den bisherigen Maßnahmen und den hier als notwendig erachteten weiteren Instrumenten, führt die Diskussion über den Abbau der kalten Progression in eine andere Richtung. Der entsprechende Vorschlag der Bundesregierung im dritten Entlastungspaket entspricht dem Sinn des Bundestagsbeschlusses vom 29. März 2012, der alle zwei Jahre einen Steuerprogressionsbericht einfordert (Deutscher Bundestag, 2012). Wolfgang Schäuble und Olaf Scholz haben seit dem Jahr 2016 dementsprechend die Eckwerte des Einkommensteuertarifs mit der Preissteigerungsrate verschoben.

Die Anpassung soll eine inflationsgetriebene automatische und damit sowohl willkürliche wie nicht kontrollierbare Steuererhöhung für die Steuerzahler:innen vermeiden. Der Einkommensteuertarif mit seinen nominalen Eckwerten wird für gegebene Realeinkommen und deren Verteilung auf ein Jahr beschlossen. Steigen die Nominaleinkommen nun in Höhe der Inflationsrate – bleiben also die Einkommen real konstant – so würden ohne Korrektur der kalten Progression die durchschnittlichen Steuersätze automatisch steigen, weil die Eckwerte des linear-progressiven Tarifs starr sind. Das heißt, dass die Steuerlast stärker ansteigt als das Einkommen und somit die reale Steuerbelastung zunimmt, ohne dass diese Steuererhöhung vom Bundestag legitimiert wurde.

Aufgrund des massiven Teuerungsimports dürfte in diesem Jahr die Inflationsrate stärker ansteigen als die Nominaleinkommen, die Realeinkommen sinken. Die kalte Progression lässt die durchschnittliche Steuerbelastung der Einkommen ansteigen. Die Besteuerung nach Leistungsfähigkeit, die durch den progressiven Einkommensteuertarif umgesetzt wird, verlangt dafür hingegen sinkende durchschnittliche Steuersätze. Um dies zu garantieren, müsste in diesem Fall der Tarif erst recht mit der Inflationsrate angepasst werden.

Die Gegner einer Korrektur der kalten Progression betonen, dass wir uns durch den Krieg in der Ukraine und den dadurch gestiegenen Energiepreisen in einer Sondersituation befänden, in der der Staat mehr Geld benötige. Die Belastung der höheren Energiepreise träfe vor allem Haushalte mit geringen Einkommen, die nicht über die Einkommensteuer entlastet werden können. Daher solle auf die Rücknahme der kalten Progression verzichtet werden und stattdessen eine Kompensation der geringen Einkommen durch Transferzahlungen erfolgen. Hierbei wird aber die Rücknahme der kalten Progression – die Verhinderung einer Steuererhöhung – mit einer Entlastungsmaßnahme verwechselt.

In der aktuellen Lage ist die Korrektur der kalten Progression ein wichtiger Pfeiler, um die Mehrbelastungen der Mittelschicht infolge der Inflation zu begrenzen. Die Tarifkorrektur von 5,8 % Inflation taxiert das Bundesfinanzministerium auf 10 Mrd. Euro Mindereinnahmen im nächsten Jahr. Tatsächlich dürfte der Steuerausfall angesichts der weiter gestiegenen Inflationsrate über 15 Mrd. Euro liegen. Grundsätzlich wäre es gut, die kalte Progression automatisch zurückzugeben, indem per Gesetz die Anpassung der Tarifzonen mit der Inflationsrate der vorangegangenen Periode erfolgt (Beznoska und Hentze, 2022).

Grundsätzlich besteht kein Widerspruch zwischen der ordnungspolitisch gebotenen – am besten automatischen – Korrektur der kalten Progression und der prozesspolitisch gebotenen Unterstützung der liquiditätsbeschränkten Haushalte mit gezielten Transfers. Beides zahlt, wenngleich aus unterschiedlichen Gründen, auf das gleiche aktuelle Ziel der Krisenpolitik ein. Die Tatsache der unterschiedlichen Begründungszusammenhänge führt aber dazu, dass kein Trade-off zwischen beiden Maßnahmen besteht. Die – dauerhaften – Steuermindereinnahmen bei der Einkommensteuer sind im laufenden Haushalten zu budgetieren, die befristeten Kosten der Transfers hingegen können als Antwort auf den exogenen Schock des Ukrainekrieges und des russischen Gasembargos unter den Ausnahmetatbestand der Schuldenbremse fallen.

The sharp rise in energy prices represents a considerable structural risk to the German economy. Deindustrialisation has become a veritable threat. The government’s economic policy needs to establish positive expectations for energy costs in the medium term through interventions in the gas and electricity markets. As in the COVID-19 pandemic, politicians should give companies generous deferral options for income and sales taxes and partially reimburse advance payments. The elimination of the cold progression does not constitute a tax relief and does not contradict the support of liquidity-weak households with targeted transfers, as both measurements are necessary for different reasons.

Prof. Dr. Michael Hüther ist Direktor des IW in Köln und Honorarprofessor an der European Business School.


Literatur

Beznoska, M. und T. Hentze (2022), Hohe Inflation entfacht kalte Progression, IW-Kurzbericht, 20/2022, Institut der deutschen Wirtschaft.
Bundesregierung (2022a), Drittes Entlastungspaket “Deutschland steht in einer schwierigen Zeit zusammen”, https://www.bundesregierung.de/breg-de/suche/drittes-entlastungspaket-2082584 (28. September 2022).
Bundesregierung (2022b), Abwehrschirm über 200 Milliarden Euro, https://www.bundesregierung.de/breg-de/aktuelles/abwehrschirm-2130944 (29. September 2022).
Deutscher Bundestag (2012), Drucksachen 17/8683, 17/9201; zu den bisher vier Steuerprogressionsberichten vgl. Bundestagsdrucksache 18/3894, 18/8183, 19/5450 und 19/22900.
Hentze, T. und M. Hüther (2022), Wirksame Hilfsprogramme in der Energiekrise. Vorschläge für mehr Liquidität in Privathaushalten und Unternehmen, IW Policy Paper, 5/2022, Institut der deutschen Wirtschaft.
Oxford Economics (2022), Europe: Winter Without Russian Gas. Can Europe avoid energy rationing (webinar), PowerPoint Presentation (https://oxfordeconomics.com).
